# Modern and ancient red fox (*Vulpes vulpes*) in Europe show an unusual lack of geographical and temporal structuring, and differing responses within the carnivores to historical climatic change

**DOI:** 10.1186/1471-2148-11-214

**Published:** 2011-07-20

**Authors:** Amber GF Teacher, Jessica A Thomas, Ian Barnes

**Affiliations:** 1 Royal Holloway University of London, Egham Hill, Egham, Surrey TW20 0EX, UK; 2 Department of Biosciences, P.O. Box 56 (Viikinkaari 9), FI-00014 University of Helsinki, Finland

**Keywords:** ancient DNA, glaciation, climate change, adaptation, carnivore

## Abstract

**Background:**

Despite phylogeographical patterns being well characterised in a large number of species, and generalised patterns emerging, the carnivores do not all appear to show consistent trends. While some species tend to fit with standard theoretical phylogeographic expectations (e.g. bears), others show little obvious modern phylogeographic structure (e.g. wolves). In this study we briefly review these studies, and present a new phylogeographical study of the red fox (*Vulpes vulpes*) throughout Europe, using a combination of ancient DNA sequences obtained from museum specimens, and modern sequences collated from GenBank. We used cytochrome *b *(250 bp) and the mitochondrial control region (268 bp) to elucidate both current and historical phylogeographical patterning.

**Results:**

We found evidence for slight isolation by distance in modern populations, as well as differentiation associated with time, both of which can likely be attributed to random genetic drift. Despite high sequence diversity (11.2% cytochrome *b*, 16.4% control region), no evidence for spatial structure (from Bayesian trees) is found either in modern samples or ancient samples for either gene, and Bayesian skyline plots suggested little change in the effective population size over the past 40,000 years.

**Conclusions:**

It is probable that the high dispersal ability and adaptability of the red fox has contributed to the lack of observable differentiation, which appears to have remained consistent over tens of thousands of years. Generalised patterns of how animals are thought to have responded to historical climatic change are not necessarily valid for all species, and so understanding the differences between species will be critical for predicting how species will be affected by future climatic change.

## Background

Many studies have looked at the phylogeographic patterns of species, primarily using mitochondrial DNA sequence comparisons, and linked the patterns seen today to severe climatic changes in the past [1,2]. During the Pleistocene (approximately 2.6 million to 12,000 years ago), Europe experienced cyclical glacial and interglacial periods, with the last glacial period ending approximately 10,000 years ago [1]. These fluctuations in climate had profound effects on species distributions, and during glacial periods temperate species in Europe are thought to have been forced south into warmer refugial areas, primarily in Iberia, Italy and the Balkans, although other smaller cryptic refugia have also been proposed [3-6]. Following the retreat of the glaciers towards the North, species were able to recolonise the now warmer and more habitable northern regions of Europe. These patterns of range contraction and expansion have shaped the genetic diversity in modern populations through a combination of genetic drift and gene flow.

Within Europe, genetic studies on terrestrial species typically find divergent clades which represent the refugial origins of the focal species during the glacial periods [3,4]. Many taxa show a deep split between Eastern and Western European clades, which likely reflects two main refugial origins in Iberia and the Italo-Balkan region [2], and generally speaking, we now have a good understanding of the routes of post-glacial expansions for various terrestrial species [1,4](Hewitt, 1999, Hewitt, 2004). Furthermore, we also have a good understanding of how these expansions occurred; theory suggests that long-distance migrants become founders of new populations [7,8], replacing the previous orthodoxy which suggested that expansions were a result of substantial genetic drift [9,10]. A loss of genetic variability typically accompanies such range expansions, with populations that are further from a refugium tending to have lower genetic diversity [3].

However, carnivores in particular seem to show discordant patterns, with some species showing strong phylogeographical structuring, whilst others show no evidence of structuring. For example, population divergence is found in brown bears in Europe and North America [2,11] and black bears in North America [12], whilst no such phylogeographical structuring is found in North American coyotes [13]. Likewise, little evidence for partitioning of haplotypes at a regional or even continental scale has been found for another carnivore, the grey wolf [14,15]. It seems probable that species with relatively high dispersal rates, combined with high adaptability to a range of habitats (i.e. high capabilities for migration and gene flow) may show less phylogeographical structuring. The swift fox, kit fox and arctic fox have also been shown to have little within-species phylogeographical structuring [16,17], however these species are refined to relatively small, specific habitat regions and so might not be expected to show structure within such restricted areas (see http://www.canids.org for distribution maps).

We present a phylogeographical study within Europe of a species that is widespread throughout the Northern hemisphere; the red fox (*Vulpes vulpes*). Previous studies on the red fox have indicated that three distinct subclades exist in North America, reflecting two recent colonisation events from North to South, and one widespread clade representing an earlier colonisation event from South to North [18]. However, an allozyme and cytochrome *b *study of modern red fox from ten locations in Southern Europe and Israel revealed little geographical structure in this limited region [19]. Our study provides a substantially wider geographical coverage of Europe, and includes ancient DNA samples dating approximately up to 40,000 years old, allowing us to add a temporal component to the study. In this study we aimed to assess the evidence for separate clades that might be associated with different refugial origins, and whether there is any evidence for a population bottleneck and subsequent expansion (as might be related to the Last Glacial Maximum). In addition, we considered the impact of genetic drift, by looking for patterns of isolation by distance and time-associated changes in haplotypes. To approach these questions, we examined variation in two mitochondrial DNA (mtDNA) genes, cytochrome *b *and the control region. Cytochrome *b *(Cyt*b*) is relatively conserved, and as such can be used to resolve deeper splits, whilst the control region harbours higher diversity and can provide higher resolution [20,21]. The use of two mtDNA gene fragments allows us to have more confidence in the results, and to help to resolve any potential ambiguities. Mitochondrial DNA is the most appropriate marker in this instance as the mutation rate is rapid enough to provide variability over the time scales in question, and recombination is very unusual, enabling straightforward interpretation of the patterns observed [21].

## Methods

### Modern data

**Figure 1:**
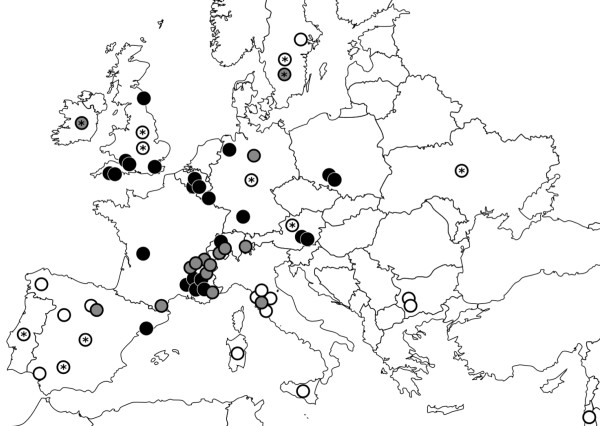
**Map to show sample locations**. White circles indicate modern cytochrome *b *data locations, grey circles indicate modern control region data locations, and black circles indicate ancient samples which have both cytochrome *b* and control region data. Those with a star '*' indicate sequences for which only the country, or region within a country, is known.

Published modern red fox sequences were collated from GenBank http://www.ncbi.nlm.nih.gov for both cytochrome *b *and the control region. Accession numbers, location information and references are listed in additional file 1, and a map is shown in Figure 1. On the occasions where multiple GenBank entries were found with an identical location and sequence, the data were collapsed into a single sequence and only one accession number is given. For incidences where a source had listed one haplotype for multiple countries, this was expanded so that there was a separate sequence for each country where this haplotype was found; multiple sites within the same country were given unique sample names.

### Ancient samples

In total, 165 European red fox bone or tooth specimens dating to the late Pleistocene and early Holocene were collected from museums and colleagues (locations, dates and donors of samples successfully amplified are listed in additional file 2). Samples were drilled onto UV-sterilised aluminium foil to ensure that there was no cross-contamination, using a clean 0.8 mm engraving cutter. Approximately 10-40 mg of bone powder was used for DNA extraction using a standard protocol for ancient bone samples [22]. Blank extractions were performed with every batch. Extractions were performed in a separate isolated laboratory, and standard precautions were taken for working with ancient DNA [23]. Six primer pairs were designed to amplify the cytochrome *b *and control region in six overlapping fragments for each marker, using Primer3 [24]. The cytochrome *b *primers were as follows (presented from 5' to 3' end): VVC1-F, GGTCCCTGCTAGGTGTATGC; VVC1-R, GTCTCGGCAGATGTGAGTGA; VVC2-F, TTGCAACAGGTCTATTTTTAGCC; VVC2-R, TAGATGCTCCGTTTGCATGT; VVC3-F, TGGCTGAATTATCCGCTACA; VVC3-R, AATTCCAATATTTCATGTTTCTATGA. The control region primers were as follows: VVD1-F, CTCCCAAAACTTGCCCTATG; VVD1-R, CTCCTGATAGAGATTATTGTAAGATT; VVD2-F, CATACTATGTTTAATCTTACAATAATCTCT; VVD2-R, CGAGCAAGGATTGATGGTTT; VVD3-F, TCCAGTAAGGGATTTATCACCA; VVD3-R, CCTGAAGTAAGAACCAGATG.

Primers were initially tested for efficacy using modern samples (donated by Dr. Peter Wandeler, Universität Zürich-Irchel and Dr. Frank Zachos, Christian-Albrechts-Universität Zu Kiel). Samples were amplified using PCR with the following reaction mixture at a total volume of 25 μl: 15.1 μl sterile double-distilled water, 2.5 μl BSA (1%), 0.4 μl each primer at 10 pmol (MWG, Germany), 0.25 μl dNTP mix (Invitrogen, UK), 0.3 μl Taq Platinum (Invitrogen, UK), 2.5 μl PCR buffer (supplied with Taq), 1 μl MgSO_4 _(supplied with Taq), 4 μl DNA. The samples were denatured at 95°C for 10 minutes followed by 40 cycles of 94°C for 30 sec, 50°C for 30 sec, and 72°C for 30 sec, followed by a final elongation step of 72°C for 7 min to complete fragment extension. Negative controls were run using blank extraction samples for each PCR run. Purification and sequencing was outsourced to Beckman-Coulter Genomics (Essex, UK). Six overlapping sequences were obtained for each gene, and all sequences were inspected manually to ensure that they were correctly scored.

### Phylogenetic analyses

Sequences were aligned using Sequencher v.4.8 (Gene Codes Corporation, Michigan, USA) and the resulting contigs consisted of 250 base pairs cytochrome *b *and 268 base pairs control region for the 35 ancient samples which successfully amplified at all 12 fragments. Modern sequence data was trimmed to cover the same regions. All ancient samples had sequence for both gene fragments, but because the modern sequences were obtained opportunistically from GenBank, we were not able to obtain sequence data for both markers for the majority of the modern samples.

We used jModeltest v.0.1.1 [25,26], and the Bayesian Information Criterion (BIC), to select the best-fit model of evolution for each gene fragment individually. A Bayesian consensus tree of the sequences was constructed using MrBayes v.3.1.2. [27]. Cytochrome *b *and control region sequences were concatenated for each individual, and for modern individuals where both gene sequences were not available the sequences were simply coded as missing data. The data was run as a partitioned dataset, with the evolutionary model for each gene (as determined by jModelTest) coded in, and allowing for each partition to evolve under a different rate. Only those models that did not include a proportion of invariant sites were considered, owing to the intra-specific nature of the data. Sampling was set to every 100 generations for 3 × 10^6 ^generations with four chains (average standard deviation of split frequencies < 0.02). We coded the chosen model of nucleotide substitution, and excluded the first 25% of samples as burn-in. The arctic fox (*Alopex lagopus*) was used as an outgroup (Accession numbers: Cytochrome *b *AY598511, control region DQ500882). To see if they provided any additional information, maximum likelihood trees were also produced using MEGA version v5.0 [28], using 500 bootstraps and the HKY model of evolution for the following datasets: (i) Cyt*b *modern samples, (ii) Cyt*b *modern+ancient samples combined, (iii) control region modern samples, (iv) control region modern+ancient samples combined, and (v) Cyt*b *and control region combined for ancient samples.

Sequences were then collapsed into haplotypes using TCS v.1.21 [29], converted to Arlequin format [30] using FaBox v.1.35 online [31], and the resulting files were used to obtain network distance connections using Arlequin v.2.000 [30]. Networks were then drawn separately for each gene fragment (as it is not possible to allow for a partitioned dataset for such analyses) using HapStar [32].

### Isolation by distance

In order to check for a geographical signal within our dataset, we correlated genetic distance to geographical distance. Pairwise nucleotide distance matrices were produced for seven datasets using MEGA v.5 [28]: (i) Cyt*b *modern samples, (ii) Cyt*b *ancient samples, (iii) Cyt*b *modern+ancient samples combined, (iv) control region modern samples, (v) control region ancient samples, (vi) control region modern+ancient samples combined, and (vii) Cyt*b *and control region combined for ancient samples. Approximate co-ordinates were identified for each site (see additional files 1 and 2) using Google Earth http://www.google.com/earth/index.html, and geographical distance matrices were created for each dataset using the Geographical Distance Matrix Generator [33]. Mantel tests were performed for each dataset using the Isolde function in GenePop on the Web [34,35], with 10,000 permutations.

### Temporal analyses

Past population dynamics can be reconstructed by estimating demographic parameters using the coalescent approach implemented in BEAST [36]. Ancient DNA can provide both temporal and phylogenetic information through the use of tip-dated sequences. In order to determine whether there was sufficient temporal signal in the data to estimate substitution rates accurately, we first conducted a date randomisation test following Firth *et al*. [37]. This test involves generating 20 datasets in which the tip-dates of each sequence are randomly shuffled. The estimates of substitution rate from these 'randomised' datasets can then be compared to the rates estimated for the 'real' dataset. The presence of sufficient temporal signal is assessed by determining whether the mean rate estimates of the real dataset fall outside the 95% highest posterior density (HPD) intervals for the rate estimates of the randomised datasets. Ancient sample ages for tip-dating were inferred through stratigraphic correlation (layer dating); any samples without dates, or with approximate or infinite ages were not included in the BEAST analysis (see additional file 2). The arctic fox outgroup *A. lagopus *was also excluded. For the BEAST analysis, models of sequence evolution for each partition were determined in jModelTest [25,26], with the nucleotide frequencies and substitution rate parameters estimated from the data. A strict clock was assumed (linked across partitions), with a constant size coalescent tree prior.

The dynamics of population size through time can be estimated using the Bayesian skyline plot in BEAST [36]. This analysis was run for 50 million generations, with parameters (described above) and genealogies sampled every 10,000 iterations and the first 20% discarded as burnin. As a number of sequences were obtained from layer-dated samples, with their ages given as ranges rather than as point estimates; uniform priors were used to model the uncertainty in these ages, rather than point-estimates (see additional file 2).

## Results

We successfully sequenced all six ancient DNA fragments for 35 samples out of the 165 collected, though some small sequence gaps were present (Figure 1, additional file 2, GenBank accession numbers JN232446-JN232515; this success rate of 21% is similar to comparable ancient DNA studies [e.g. [38,39]]. The successful samples represent 24 localities and 9 countries. We also obtained 38 modern cytochrome *b *sequences from GenBank, from 22 localities in 12 countries, and 39 control region sequences from 15 localities in 7 countries. Sequence diversity was high within the datasets; 26 haplotypes were identified in the cytochrome *b *sequences (modern + ancient), and 47 haplotypes in the control region sequences. We identified 28 (11.2% of bases) and 44 (16.4% of bases) polymorphic sites in the cytochrome *b *and control region fragments respectively.

### Phylogenetic analyses

The jModelTest analyses determined that the least complex model of evolution with the best fit to the cytochrome *b *data was the HKY model (Ti/Tv = 6.4802, unequal base frequencies freqA = 0.289, freqC = 0.231, freqG = 0.156, freqT = 0.324), and for the control region was the TIM2+G model (unequal base frequencies freqA = 0.295, freqC = 0.241, freqG = 0.137, freqT = 0.328, unequal substitution rates r_AC _= 0.244, r_AG _= 5.916, r_AT _= 0.244, r_CG _= 1.000, r_CT _= 2.133, r_GT _= 1.000, scaled to r_GT_, and gamma shape distribution = 0.130).

**Figure 2:**
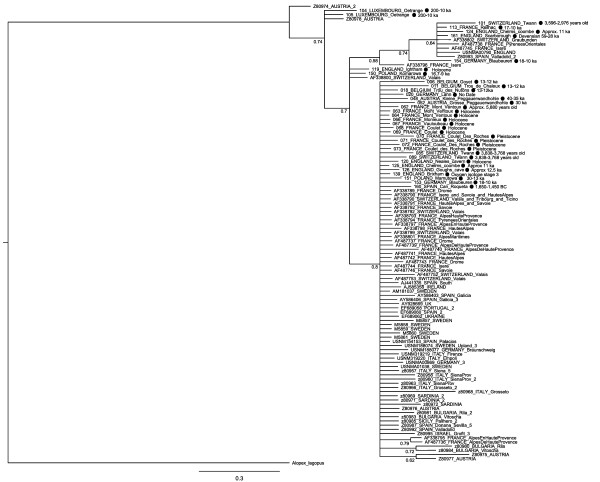
**Bayesian tree**. This tree was produced using a partitioned dataset including cytochrome *b *and the control region for all ancient samples and modern samples. Many modern samples only had one gene represented, and so the other gene was coded as missing data. Ancient samples are marked with a black circle and an approximate date, whilst all others are modern samples.

The Bayesian tree (Figure 2) showed no obvious geographically or temporally based clades, despite reasonable sequence diversity (see above). Although some smaller clades can be seen, the posterior probabilities do not show particularly high support, all but one (Luxembourg) of the locations found in the smaller clades are also found in the larger main clade (England, France, Germany, Spain, Austria, Poland and Switzerland), and it is of note that the most basal sample is not an ancient sample, but a modern Austrian sample. The maximum likelihood trees were less informative than the Bayesian tree, with very low bootstrap support values (typically < 60) and consequently have not been included. Haplotype networks for cytochrome *b *and the control region (Additional files 3 and 4) also show no indication of haplotype clustering, and both networks show a star-like construction, which is consistent with gradual population expansion. Furthermore, there appears to be no clustering by sample age; ancient samples do not seem to cluster together, nor do they appear only at the centre of the networks (as might indicate an expansion event from the ancient samples), nor do they appear only at the edges of the networks (as could indicate a bottleneck). Specifically, it is worth noting that the oldest samples (Austria 30 ka, Austria 35-40 ka and England 59-28 ka) do not cluster together.

### Isolation by distance

We detected a slight but statistically significant signal of positive isolation by distance in one dataset: (iv) control region modern samples (R^2 ^= 0.048, *p*(one-tailed) = 0.026). No evidence for isolation by distance was detected for the remaining datasets: (i) Cyt*b *modern samples, (ii) Cyt*b *ancient samples, (iii) Cyt*b *modern+ancient samples combined, (v) control region ancient samples, (vi) control region modern+ancient samples combined, and (vii) Cyt*b *and control region combined for ancient samples.

### Temporal analyses

**Figure 3:**
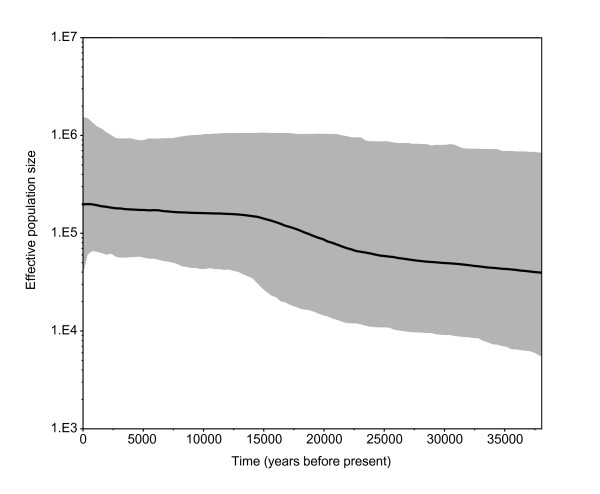
**Bayesian skyline plot showing red fox effective population size through time**. Median estimates are shown by the solid black line, and 95% HPD intervals are indicated by the surrounding grey area.

The models of sequence evolution for the BEAST analysis were determined by jModelTest as the HKY model for the cytochrome *b *data and the TIM1+G model for the control region (this differs slightly from the cytochrome *b *model used for the phylogeny, as a reduced dataset comprising of only dated samples was used for the BEAST analysis; see additional file 2). The date randomisation test indicated there was sufficient temporal signal in the data to estimate substitution rates, with the mean rate estimates for the real datasets falling outside the 95% HPD intervals of the date-randomised datasets (see additional file 5). The mean substitution rate was estimated as 2.987 × E-7 substitutions/site/year. The Bayesian skyline plot (Figure 3) indicates a marginal increase in population size over the last 40,000 years, compatible with the hypothesis that there was no substantial change in red fox population size during the last glacial maximum and into the Holocene.

## Discussion

We detected signals of isolation by distance (in modern control region samples), as well as differentiation associated with time, but we did not detect any evidence for a historical bottleneck or for phylogeographical structure within Europe. Furthermore, this lack of structure appears to have been consistent over tens of thousands of years, though it is also possible that structure existed historically but that we were unable to detect it. Our results imply that low levels of random genetic drift have caused slight changes over space and time, but that fox populations in Europe were most likely not forced into glacial refugia, instead managing to survive the glaciations as a single large interbreeding population.

### Isolation by distance and time

Evidence for isolation by distance was only detected in the modern control region sequences; however it is probable that the modern cytochrome *b *data simply did not have the resolution to detect this pattern. No isolation by distance was detected in any of the ancient datasets tested (or the combined modern and ancient datasets for either gene), most likely because while the modern dataset represents a snapshot in time, the ancient samples come from a very wide time range, and so patterns of isolation by distance may be obscured. The degree of isolation by distance detected most likely represents the effects of random genetic drift.

Date randomisation tests revealed differentiation associated with time, i.e. there is a detectable correlation between genetic distance and temporal distance.

Several haplotypes are shared between modern and ancient samples (despite several tens of thousands of years separating some of the samples), and the ancient samples do not form a separate or basal clade, providing supporting evidence that changes over time have not been severe. Again, these changes over time can probably be attributed to random genetic drift.

### Lack of phylogenetic structure or evidence for a bottleneck

Phylogenetic studies frequently detect separate lineages in Europe which reflect population bottlenecks, isolation in glacial refugia, and subsequent recolonisation.

However, we found no evidence for separate lineages within our data, and our Bayesian skyline plots indicate a relatively constant population size. Furthermore, there is no evidence of a severe loss of unique haplotypes dating to before the Last Glacial Maximum (LGM, circa 23,300 to 27,800 years ago [40]) as might be expected from a bottleneck (Figure 3). These results combined suggest that the red fox was present in Europe throughout the glaciation, rather than being forced into glacial refugia with re-emergence and population expansion during the warmer periods. However, it is also possible that historical population structure may have existed but is undetected in our study, perhaps due to a relatively small sample size from each time period. Undetected historical phylogeographic structure could have since been reduced by gene flow, leading to a lack of structure in modern samples.

Our findings provide strong support for those of previous smaller scale studies, which found little genetic structuring in modern red foxes in the South of Europe and Israel [19], or in France [41]. Our findings do however appear to be contradictory to a previous study in the red fox which found some evidence for phylogeographic structuring in North America [18], however it is probable that this difference reflects the very different histories of the two regions. American red foxes are thought to have been isolated for a long time (during the Wisconsin glaciation, between 110,000 and 10,000 BP) in two disjunct refugia [42,43], However, there is no evidence for such a prolonged glaciation and subsequent isolation of populations in Europe; as such, the results of our study and that of Aubry *et al*. [18] are not necessarily contradictory to each other. It is probable that two characteristics of the red fox may have contributed to the lack of structuring found in our study; a high dispersal rate, and high adaptability to different habitats.

### Dispersal and adaptability

Dispersal ability is expected to have a strong effect on genetic differentiation, as high dispersal can mean high gene flow and thus lower levels of differentiation. The red fox has a highly flexible social system, with both dispersing and non-dispersing males and females [44]. Those that do disperse are known to do so over large distances, for example a mark-recapture study showed that a single fox, tagged as a pup, had dispersed 302 km in North Dakota [45]. Mean dispersal distances of tagged pups were quoted within the same study as 35 km for males and 25 km for females, with the majority of recoveries of tagged individuals occurring by 1 year. Another species that shows little phylogeographic structure, the grey wolf, also has a very high dispersal rate, with movements of over 1,000 km recorded for an individual [14], and coyotes also show low structuring and high dispersal (50-100 km) [13,46,47]. However, dispersal rates alone are not enough to explain why some carnivores show phylogeographic structure whilst others do not. The degree of specialisation versus adaptability is also likely to play a key role. The red fox is highly adaptable, as exemplified by a range that encompasses almost the entire Northern hemisphere, including regions above the arctic circle, and South as far as Yemen and Northern India [48]. Sub-fossil assemblages indicate that between 38-25 BC (i.e. before the LGM), the distribution of the red fox is likely to have covered central Europe, and at least as far north as Southern England [49]. A radiocarbon date from the UK of 28,641 ± 468 cal BP [50] confirms that this species was present in Northern Europe up to a very short time before the LGM. Following the LGM, and the retreat of the glaciers, the red fox is thought to have quickly returned to central Europe, and fossils have been found within assemblages that date to 15,500 BP in northern Germany [51], and from 16,000-15,500 BP in south-eastern Germany [52]. When this information is combined, it seems that the red fox was likely only forced into the warmer South for a short period during the LGM. Iberia, Italy, Southern France, the Balkans, the Carpathians and the Crimean peninsula have all been suggested as possible glacial refugia for the red fox, based on the sub-fossil record [reviewed in [49,53]]. However, there are records of this species being present in Spain, Portugal, France, Italy, Austria, Hungary, Czech Republic, Slovakia and Slovenia during the LGM, indicating a wide and continuous range during this period [53]. This information from the fossil record, and circumstantial evidence that this species highly adaptable and able to survive in sub-arctic conditions today, both support our genetic findings in indicating that the red fox may not have been strictly restricted to refugia during the European glaciations.

Differences in dispersal ability and adaptability can cause very different outcomes, as respectively exemplified in the following two species studies, brown bears and artic fox. In a study on brown bears (*Ursus arctos*), Valdiosera *et al*. [38] showed that although bears in modern Europe do show differentiation into major lineages, this pattern breaks down in ancient samples. The authors concluded that this highly adaptable species, with its very flexible diet, was able to survive across a large area in Southern Europe during the glaciations, as we are suggesting was also the case for the red fox. However, in contrast our findings for the red fox, brown bear lineage separations occurred later, most likely due to low dispersal distances and decreasing population sizes. A rather different pattern can be found within the Arctic fox however, which is a relatively specialist species but with good dispersal abilities. A recent phylogeographic study on the arctic fox (*Alopex lagopus*) examined the haplotypes that were present in Europe during the Pleistocene and compared these with the present-day haplotypes. This study indicated that arctic fox populations from mid-latitude Europe became extinct as the climate warmed at the end of the last ice-age, and that this species did not track its arctic habitat as it shifted North to Scandinavia; the present-day Scandinavian arctic foxes are thought to originate from a later expansion event from Siberia [39]. This is strongly in contrast to our findings from red fox, which indicate little change in haplotypes present despite substantial climatic change.

## Conclusions

To the best of our knowledge, this is the first time that a study has found such a spatially and temporally wide-scale lack of phylogeographic structure in a terrestrial species within Europe. Our study adds to a collection of phylogeographical studies on carnivores, which when looked at together indicate that there may be a difference in response to historical climatic change between species with differing traits such as dispersal rates and adaptability. It seems likely that the high dispersal abilities, and adaptability to a wide habitat range, has led to the lack of observable differentiation in the red fox, and it is possible that such traits mean that this species may respond well to future climatic changes. However this also raises our awareness that quite similar species can respond very differently.

A comprehensive study into the life-history traits of carnivores, including their niche breadth, dispersal abilities, home range, body size, and responses to historical climatic change would perhaps be enlightening, allowing a deeper understanding of the traits that determine how a species is likely to be affected by changes in habitat availability.

Realising that generalised patterns are unlikely to be applicable to all species, and understanding the differences between how species responded to historical climatic changes may help us to create better predictions of how animal species will be affected by climate changes in the future.

## Authors' contributions

AGFT, JAT and IB conceived the study, AGFT collected the samples and performed the molecular work, AGFT and JAT analysed the data, AGFT wrote the manuscript, AGFT, JAT and IB contributed and finalised subsequent drafts.

## Supplementary Material

Additional file 1 **Modern sequence information**. GenBank accession numbers, location information, and number of sequences used in modern phylogeny. Click here for file

Additional file 2 **Ancient sample information**. Ancient samples, locations, museum codes, sample donors, and dating information, including the approximations of the dates used for the BEAST analysis. Click here for file

Additional file 3 **Cytochrome *b *haplotype network**. White circles represent modern sequences, black circles represent ancient sequences, and split circles represent haplotypes present both in ancient and modern samples. Each haplotype has a unique number, and the country of origin is marked nearby, together with the number of sequences represented in brackets. Details for the central haplotype are shown to the side. For ancient samples, where available the approximate date of the sample is also given in brackets; those marked with a star '*' are dated by dendrochronology, the one sample marked with two stars '**' is radiocarbon dated, and all others are contextual dates. Click here for file

Additional file 4 **Control region haplotype network**. White circles represent modern sequences, black circles represent ancient sequences, and split circles represent haplotypes present both in ancient and modern samples. Each haplotype has a unique number, and the country of origin is marked nearby, together with the number of sequences represented in brackets. For ancient samples, where available the approximate date of the sample is also given in brackets; those marked with a star '*' are dated by dendrochronology, the one sample marked with two stars '**' is radiocarbon dated, and all others are contextual dates. Click here for file

Additional file 5 **Marginal probability distributions for real and randomised datasets**. Marginal probability distributions for real and randomised datasets, with the mean substitution rate of 2.987 substitutions per site per year indicated for the real dataset. Real datasets are indicated in black, randomised are in colour. Click here for file
