# Supplementary material for: Modern and ancient red fox (Vulpes vulpes) in Europe show an unusual lack of geographical and temporal structuring, and differing responses within the carnivores to historical climatic change
Source: BMC Evol Biol. 2011 Jul 20;11:214. doi: 10.1186/1471-2148-11-214 (PMC3154186; doi:10.1186/1471-2148-11-214)
Supplement: Additional file 1 — Modern sequence information. GenBank accession numbers, location information, and number of sequences used in modern phylogeny. [file 1471-2148-11-214-S1.DOC]

**Additional file 1**

| **Cytochrome b** | | |  | **Control region** | | |  |
| --- | --- | --- | --- | --- | --- | --- | --- |
| **Accession number** | **Location** | **n(sequences)** | **Approx co-ordinates** | **Accession number** | **Location** | **n(sequences)** | **Approx co-ordinates** |
| z80974 | Austria | 2 | n/a | af487736 | France, Alpes de Haute Provence | 1 | 44°13'N 6°24'E |
| z80975 | Austria | 1 | n/a | af338795 | France, Alpes de Haute Provence | 1 | 44°13'N 6°24'E |
| z80977 | Austria | 1 | n/a | af487740 | France, Alpes de Haute Provence | 1 | 44°13'N 6°24'E |
| z80978 | Austria | 1 | n/a | af487739 | France, Alpes de Haute Provence | 1 | 44°13'N 6°24'E |
| z80979 | Austria | 1 | n/a | af338793 | France, Alpes de Haute Provence | 1 | 44°13'N 6°24'E |
| z80980 | Bulgaria, Rila | 1 | 42°7'N 23°8'E | af338797 | France, Alpes de Haute Provence | 1 | 44°13'N 6°24'E |
| z80983 | Bulgaria, Vitoscha | 1 | 42°38'N 23°16'E | af338801 | France, Alpes Maritimes | 1 | 43°56'N 7°10'E |
| z80984 | Bulgaria, Vitoscha | 1 | 42°38'N 23°16'E | af338789 | France, Drome | 1 | 44°43'N 5°13'E |
| z80981 | Bulgaria, Rila | 1 | 42°38'N 23°16'E | af487737 | France, Drome | 1 | 44°43'N 5°13'E |
| usnma00790 | England | 1 | n/a | af487742 | France, Drome | 1 | 44°43'N 5°13'E |
| usnma00869 | Germany | 3 | n/a | af338798 | France, Hautes Alpes | 1 | 44°36'N 6°17'E |
| z80995 | Israel, Grofit | 3 | 29°56'N 35°3'E | af487742 | France, Hautes Alpes | 1 | 44°36'N 6°17'E |
| usnm319220 | Italy, Empoli | 1 | 42°43'N 10°56'E | af487741 | France, Hautes Alpes | 1 | 44°36'N 6°17'E |
| usnm319219 | Italy, Firenze | 1 | 43°46'N 11°15'E | af338791 | France, Hautes Alpes and Savoie | 1 | Multiple locations-see other entries. |
| z80966 | Italy, Grosseto | 2 | 42°45'N 11°6'E | af338796 | France, Isere | 1 | 44°59'N 5°55'E |
| z80968 | Italy, Grosseto | 1 | 42°45'N 11°6'E | af487745 | France, Isere | 1 | 44°59'N 5°55'E |
| z80958 | Italy, Siena | 1 | 43°18'N 11°19'E | af487744 | France, Isere | 1 | 44°59'N 5°55'E |
| z80957 | Italy, Siena | 5 | 43°18'N 11°19'E | af338790 | France, Isere, Savoie, Hautes Alpes | 1 | Multiple locations-see other entries. |
| z80960 | Italy, Siena | 2 | 43°18'N 11°19'E | af487738 | France, Pyrenees Orientales | 1 | 42°36'N 2°33'E |
| z80963 | Italy, Siena | 1 | 43°18'N 11°19'E | af338794 | France, Pyrenees Orientales | 1 | 42°36'N 2°33'E |
| ef689058 | Portugal | 2 | n/a | af338792 | France, Savoie | 1 | 45°29'N 6°28'E |
| z80969 | Sardinia | 2 | 40°3'N 9°3'E (central Sardinia) | af487746 | France, Savoie | 1 | 45°29'N 6°28'E |
| z80971 | Sardinia | 2 | 40°3'N 9°3'E (central Sardinia) | usnm188077 | Germany, Braunschweig | 1 | 52°15'N 10°31'E |
| z80972 | Sardinia | 1 | 40°3'N 9°3'E (central Sardinia) | aj585358 | Ireland | 1 | n/a |
| z80985 | Sicily, Palermo | 2 | 38°6'N 13°21'E | usnm319220 | Italy, Empoli | 1 | 42°43'N 10°56'E |
| ef689060 | Spain | 2 | n/a | usnm154153 | Spain, Palacios | 1 | 42°53'N 6°35'W |
| usnm154153 | Spain | 1 | n/a | m5858 | Sweden | 1 | n/a |
| aj441335 | Spain South | 1 | n/a | m5859 | Sweden | 1 | n/a |
| z80987 | Spain, Donana Sevilla | 5 | 36°53'N 6°23'W | am181037 | Sweden | 1 | n/a |
| ay586403 | Spain, Galicia | 1 | 42°34'N 8°8'W | m5860 | Sweden | 1 | n/a |
| ay586406 | Spain, Galicia | 3 | 42°34'N 8°8'W | m5861 | Sweden | 1 | n/a |
| z80992 | Spain, Valladolid | 1 | 41°39'N 4°43'W | m5857 | Sweden | 1 | n/a |
| z80993 | Spain, Valladolid | 2 | 41°39'N 4°43'W | af338802 | Switzerland, Graubunden | 1 | 46°39'N 9°34'e |
| am181037 | Sweden | 1 | n/a | af338799 | Switzerland, Valais | 1 | 46°11'N 7°34'E |
| usnma01038 | Sweden | 1 | n/a | af487753 | Switzerland, Valais | 1 | 46°11'N 7°34'E |
| usnm188074 | Sweden, Upland | 3 | 59°51'N 17°38'E (uppsala) | af338800 | Switzerland, Valais | 1 | 46°11'N 7°34'E |
| ay928669 | UK | 1 | n/a | af338792 | Switzerland, Valais | 1 | 46°11'N 7°34'E |
| ef689062 | Ukraine | 1 | n/a | af487752 | Switzerland, Valais | 1 | 46°11'N 7°34'E |
|  |  |  |  | af338790 | Switzerland, Valais and Fribourg | 1 | Multiple locations-see other entries  (Fribourg: 46°48'N 7°8'E) |
