# Supplementary material for: Modern and ancient red fox (Vulpes vulpes) in Europe show an unusual lack of geographical and temporal structuring, and differing responses within the carnivores to historical climatic change
Source: BMC Evol Biol. 2011 Jul 20;11:214. doi: 10.1186/1471-2148-11-214 (PMC3154186; doi:10.1186/1471-2148-11-214)
Supplement: Additional file 2 — Ancient sample information. Ancient samples, locations, museum codes, sample donors, and dating information, including the approximations of the dates used for the BEAST analysis. [file 1471-2148-11-214-S2.DOC]

**Additional file 2**

| **Lab code** | **Country** | **Site** | **Museum code** | **Sample donor** | **Date** | **Approximation for BEAST analysis** | **Approximate co-ordinates** | **Cytb accession number** | **Control region accession number** |
| --- | --- | --- | --- | --- | --- | --- | --- | --- | --- |
| AT006 | Belgium | Goyet | 2830-1 | Collection Dupont, Belgium | 12-13ka | 12500*** | 50°26'N 5°0'E | JN232446 | JN232493 |
| AT011 | Belgium | Trou de Chaleux | 2623-6 | Collection Dupont, Belgium | 12-13ka | 12500*** | 50°13'N 4°57'E | JN232447 | JN232496 |
| AT018 | Belgium | Trou des Nutons, Furfooz | 2560-6 | Collection Dupont, Belgium | 12-13ka | 12500*** | 50°13'N 4°57'E | JN232448 | JN232500 |
| AT020 | Germany | Steeten/Lahn | MB.Ma.1386 | Oliver Hampe, Berlin | No date | n/a | 52°49'N 7°37'E | JN232449 | JN232505 |
| AT048 | Austria | Kleine Peggauerwandhöhle | KP21/3 | Florian Fladerer, Vienna | 35-40ka | 37500*** | 47°12'N 15°22'E | JN232450 | JN232494 |
| AT052 | Austria | Große Peggauerwandhöhle | GP31/1 | Florian Fladerer, Vienna | 30ka | 30000 | 47°12'N 15°22'E | JN232451 | JN232481 |
| AT062 | France | Mont Ventoux 2 | None | Evelyne Cregut, Requien Museum Avignon | Approx 5,880 years old | 5880 | 44°10'N 5°17E | JN232452 | JN232506 |
| AT063 | France | Mont Ventoux 2 | None | Evelyne Cregut, Requien Museum Avignon | Holocene | 5700*** | 44°10'N 5°17E | JN232453 | JN232510 |
| AT064 | France | Mont Ventoux 2 | None | Evelyne Cregut, Requien Museum Avignon | Holocene | 5700*** | 44°10'N 5°17E | JN232454 | JN232487 |
| AT066 | France | Aven des Planes, Monieux | None | Evelyne Cregut, Requien Museum Avignon | Holocene | 5700*** | 44°4'N 5°21'E | JN232455 | JN232484 |
| AT067 | France | Vauloubeau | None | Evelyne Cregut, Requien Museum Avignon | Holocene | 5700*** | 43°56'N 5°23'E | JN232456 | JN232486 |
| AT068 | France | Aven du Chat, Coulet | None | Evelyne Cregut, Requien Museum Avignon | Holocene | 5700*** | 44°10'N 6°9'E | JN232457 | JN232512 |
| AT069 | France | Aven du Chat, Coulet | None | Evelyne Cregut, Requien Museum Avignon | Holocene | 5700*** | 44°10'N 6°9'E | JN232458 | JN232503 |
| AT070 | France | Coulet des Roches | Carre J3, No. 170 | Evelyne Cregut, Requien Museum Avignon | Pleistocene, depth ±167.8 | n/a | 44°2'N 5°23'E | JN232459 | JN232495 |
| AT071 | France | Coulet des Roches | Carre K4, No. 14 | Evelyne Cregut, Requien Museum Avignon | Pleistocene, depth ±230.4 | n/a | 44°2'N 5°23'E | JN232460 | JN232515 |
| AT072 | France | Coulet des Roches | Carre I3, No.43 | Evelyne Cregut, Requien Museum Avignon | Pleistocene, depth ± 167.8 | n/a | 44°2'N 5°23'E | JN232461 | JN232504 |
| AT073 | France | Coulet des Roches | Carre I3, No.38 | Evelyne Cregut, Requien Museum Avignon | Pleistocene, depth ±156.4 | n/a | 44°2'N 5°23'E | JN232462 | JN232514 |
| AT085 | Switzerland | Twann | 59 | Marc Nussbaumer, Berne Nat Hist Museum | 3,838-3,768 years old (denrochronology) | 3800*** | 47°5'N 7°9'E | JN232463 | JN232499 |
| AT089 | Switzerland | Twann | 328 | Marc Nussbaumer, Berne Nat Hist Museum | 3,838-3,768 years old (denrochronology) | 3800*** | 47°5'N 7°9'E | JN232464 | JN232509 |
| AT101 | Switzerland | Twann | 229 | Marc Nussbaumer, Berne Nat Hist Museum | 3,596-2,976 years old (dendrochronology) | 3300*** | 47°5'N 7°9'E | JN232465 | JN232482 |
| AT104 | Luxembourg | Oetrange | None | Christine Argot and Pascal Tassy, MNHN Paris | 200-10ka | n/a | 49°35'N 6°15'E | JN232466 | JN232498 |
| AT105 | Luxembourg | Oetrange | None (1937-294) | Christine Argot and Pascal Tassy, MNHN Paris | 200-10ka | n/a | 49°35'N 6°15'E | JN232467 | JN232488 |
| AT113 | France | Reilhac | None | Christine Argot and Pascal Tassy, MNHN Paris | 17-10ka | 13500*** | 45°44'N 1°38'E | JN232468 | JN232491 |
| AT119 | England | Ightham | d | Andy Currant, NHM London | Holocene | 5700*** | 51°17'N 0°17'E | JN232469 | JN232508 |
| AT120 | England | Neale's cavern, Devon | M22124 | Andy Currant, NHM London | Holocene | 5700*** | 55°22'N 3°31'E | JN232470 | JN232501 |
| AT124 | England | Chelm's Coombe, Cheddar | A8 | Andy Currant, NHM London | Approx 11ka | 11000 | 51°16'N 2°46'E | JN232471 | JN232483 |
| AT125 | England | Chelm's Coombe, Cheddar | AIX | Andy Currant, NHM London | Approx 11ka | 11000 | 51°16'N 2°46'E | JN232472 | JN232513 |
| AT126 | England | Gough's Cave, Somerset | M51599 | Andy Currant, NHM London | Pleistocene, late devensian, layer 11, approx 12.5ka | 12500 | 51°17'N 2°38'E | JN232473 | JN232485 |
| AT139 | England | Brixham (near Torquay) | 48906 | Andy Currant, NHM London | Oxygen isotope stage 3 - Pinhole mammal assemblage | n/a | 50°23'N 3°30E | JN232474 | JN232511 |
| AT150 | Poland | Komarowa cave, Poland | PL-1-KOM | Adam Nadachowski and Mr Grzegorz Lipecki | Layer B, 7 dates (OSL, TL and C14) between 16.7-9ka* | 12800*** | 50°40'N 17°55'E | JN232475 | JN232489 |
| AT151 | Poland | Mamutowa cave, Poland | PL-2-MM | Adam Nadachowski and Mr Grzegorz Lipecki | Layer 2: several C14 dates range from 13-30ka** | 21500*** | 50°4'N 19°51'E | JN232476 | JN232497 |
| AT153 | Germany | Brillenhöhle, Blaubeuren | X | Henriette Obermaier, Munchen | 18-10ka | 14000*** | 48°24'N 9°47'E | JN232477 | JN232502 |
| AT154 | Germany | Brillenhöhle, Blaubeuren | XI | Henriette Obermaier, Munchen | 18-10ka | 14000*** | 48°24'N 9°47'E | JN232478 | JN232490 |
| AT160 | Spain | Can Roqueta II | Structure 753, level 1 | Jordi Lorenzo, Barcelona | 1,650-1,450 cal BC | 1500*** | 41°32'N 2°6'E | JN232479 | JN232507 |
| AT161 | UK | Scalby Bay, Scarborough | M82957 | Andy Currant, NHM London | Devensian 59-28ka | n/a | 54°16'N 0°24'E | JN232480 | JN232492 |

* These dates were published in the book: Nadachowski A, Zarski M, Urbanowski M, Wojtal P, Miekina B, Lipecki G, Ochman K, Krawczyk M, Jakubowski G, Tomek T: *Late Pleistocene environment of the Czestochowa Upland (Poland) reconstructed on the basis of faunistic evidence from archaeological cave sites*. Krakow: Institute of Systematics and Evolution of Animals, Polish Academy of Sciences; 2009.

** These dates were published in the book: Wojtal P: *Zooarcheological studies of the Late Pleistocene sites in Poland*. Krakow: Institute of Systematics and Evolution of Animals, Polish Academy of Sciences; 2007.

*** Indicates uniform priors were placed on tip-date range estimates.
