# Supplementary figures and images for: Modern and ancient red fox (Vulpes vulpes) in Europe show an unusual lack of geographical and temporal structuring, and differing responses within the carnivores to historical climatic change
Source: BMC Evol Biol. 2011 Jul 20;11:214. doi: 10.1186/1471-2148-11-214 (PMC3154186; doi:10.1186/1471-2148-11-214)

**Additional file 3**

**
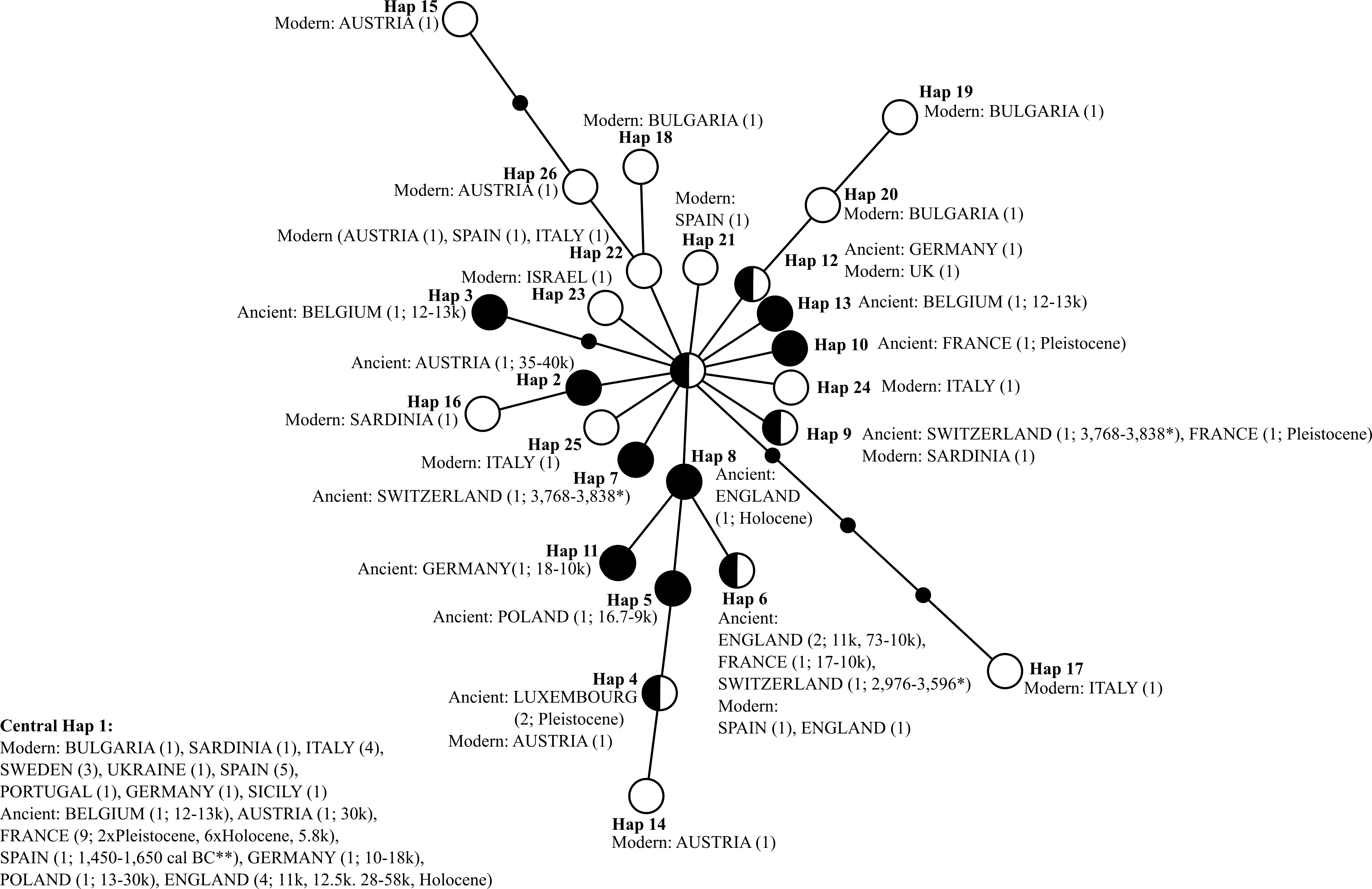
**

Supplement: Additional file 3 — Cytochrome b haplotype network. White circles represent modern sequences, black circles represent ancient sequences, and split circles represent haplotypes present both in ancient and modern samples. Each haplotype has a unique number, and the country of origin is marked nearby, together with the number of sequences represented in brackets. Details for the central haplotype are shown to the side. For ancient samples, where available the approximate date of the sample is also given in brackets; those marked with a star '*' are dated by dendrochronology, the one sample marked with two stars '**' is radiocarbon dated, and all others are contextual dates. [file 1471-2148-11-214-S3.DOC]

**Additional file 4**

**
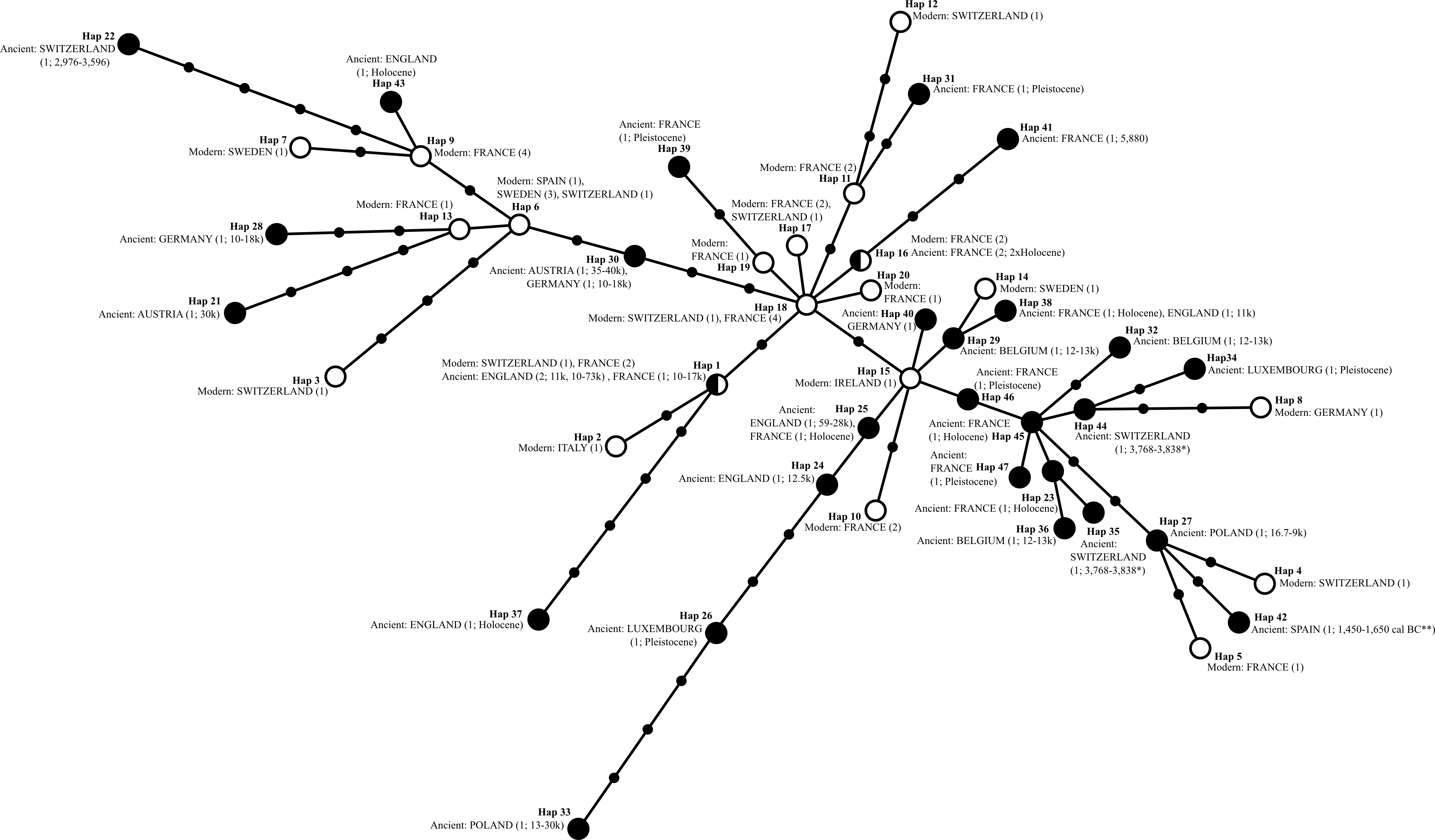
**

Supplement: Additional file 4 — Control region haplotype network. White circles represent modern sequences, black circles represent ancient sequences, and split circles represent haplotypes present both in ancient and modern samples. Each haplotype has a unique number, and the country of origin is marked nearby, together with the number of sequences represented in brackets. For ancient samples, where available the approximate date of the sample is also given in brackets; those marked with a star '*' are dated by dendrochronology, the one sample marked with two stars '**' is radiocarbon dated, and all others are contextual dates. [file 1471-2148-11-214-S4.DOC]

**Additional file 5**


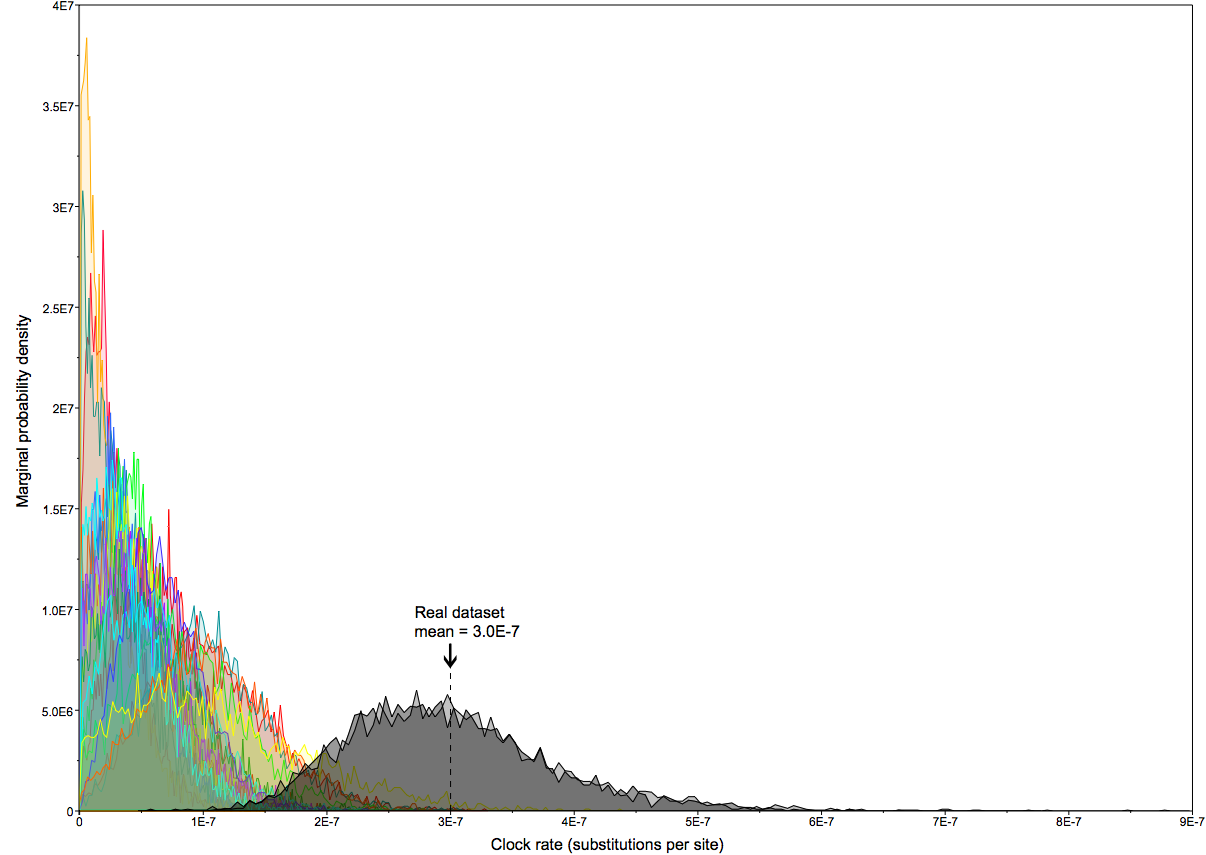

Supplement: Additional file 5 — Marginal probability distributions for real and randomised datasets. Marginal probability distributions for real and randomised datasets, with the mean substitution rate of 2.987 substitutions per site per year indicated for the real dataset. Real datasets are indicated in black, randomised are in colour. [file 1471-2148-11-214-S5.DOC]
